# Risk factors associated with tuberculosis recurrence in South Korea determined using a nationwide cohort study

**DOI:** 10.1371/journal.pone.0268290

**Published:** 2022-06-16

**Authors:** Hin Moi Youn, Moon-Kyung Shin, Dawoon Jeong, Hee-Jin Kim, Hongjo Choi, Young Ae Kang

**Affiliations:** 1 Department of Family Medicine and Primary Care, University of Hong Kong, Hong Kong, China; 2 Institute of Health Services Research, Yonsei University, Seoul, Republic of Korea; 3 Division of Pulmonary and Critical Care Medicine, Department of Internal Medicine, Severance Hospital, Yonsei University College of Medicine, Seoul, Republic of Korea; 4 Korean National Tuberculosis Association, Seoul, Republic of Korea; 5 Department of Preventive Medicine, Konyang University College of Medicine, Daejeon, Republic of Korea; 6 Institute of Immunology and Immunological Disease, Yonsei University College of Medicine, Seoul, Republic of Korea; Inha University Hospital, REPUBLIC OF KOREA

## Abstract

**Objective:**

Prevention of tuberculosis (TB) recurrence is an important issue in TB control. South Korea, a country with a high average income, has been challenged with an intermediate burden of TB. We aimed to estimate the TB recurrence rate after successful completion of the first anti-TB chemotherapy, and to identify the risk factors for the TB recurrence by focusing on co-morbidities and behavioral factors.

**Methods:**

This is a population-based cohort study using data from the National Health Insurance (NHI) database between 2002 and 2013. Newly diagnosed TB patients were identified using the classification of disease codes and prescription records. Final analytical subjects included people who successfully completed the first anti-TB chemotherapy. The primary outcome measure was recurrent TB 6-month after the first treatment completion. A set of associated risk factors, including demographic characteristics, co-morbidities, and health behavior factors were analyzed using Cox regression analysis.

**Results:**

Among 5,446 TB patients, 2,226 (40.1%) completed the first anti-TB treatment. During the follow-up period, 150 (6.7%) patients had TB recurrence, and the crude recurrent rate was 22.6 per 1000 person-years. The majority of recurrence cases (89%) occurred within the first 2-year period. The major findings show that participants who are male (adjusted HR (aHR) = 1.81, at a 95% CI, range: 1.11–2.94), older in age (aHR = 1.07, at a 95% CI, range: 1.00–1.14), have a lower income (aHR = 1.96, at a 95% CI, range: 1.10–3.48) and who are underweight (aHR = 1.92, at a 95% CI, range 1.15–3.20) were at higher risks for TB recurrence.

**Conclusion:**

People who have risk factors for recurrent TB need to improve treatment compliance through more effective TB management, and follow-up observation for one or two years after the treatment completion.

## 1. Introduction

Even after the successful treatment of tuberculosis (TB), people with a history of TB are at higher risk for its recurrence [1]. Tuberculosis disease among previously treated individuals (recurrent TB) constitutes approximately 7% of incident global cases [2]. People with recurrent TB also have poorer outcomes, including lower treatment completion and higher mortality [3, 4]. In addition, recurrent TB shows a higher rate of drug resistance. The percentage of rifampin-resistant/multi-drug resistant TB (RR/MDR-TB) is higher in previously treated TB cases than in new cases, and the risk ratio is 4.2 when comparing recurrence to new cases [2]. Thus, recurrent TB cases can pose a challenge to dedicated programs and recurrence rates are an important indicator of the effectiveness of TB control programs.

After successful treatment, the incidence of recurrent TB across 145 countries was estimated 2.26 per 100 person-years (at a 95% CI, range: 1.88–2.72), ranged from 0.05 to 29.52 [5]. This reflects the heterogeneity of study designs, the definition of recurrence, local TB incidence and the study populations analyzed. In addition, a variety of risk factors for recurrence have been shown in previous studies, including medical co-morbidities, behavioral factors, socio-economic status and bacteriologic factors [6–8]. Elucidating factors associated with recurrent TB could help control programs and clinical providers to recognize more vulnerable populations at a greater risk for recurrence. Furthermore, it can allow them to explore ways of minimizing avoidable risks by focusing on post-treatment follow-up.

South Korea is a high-income country with an intermediate TB burden. With the successful implementation of a national TB control program, incidence has decreased, but was still a considerable 49.4 per 100,000 persons in 2020 [9]. According to the TB surveillance system record, people who were re-treated for TB accounted for 14.6% of the total cases in 2020, which was a decrease from 25.3% in 2001 [9]. With less than 15,000 people with HIV infection in 2020, the prevalence of HIV infection in South Korea is relatively low [10]. However, non-communicable diseases, such as diabetes, are on the rise [11]. Additionally, a rapid aging population, with an increasing proportion of TB in the elderly, is an obstacle in TB control programs in South Korea. In this situation, longitudinal studies on TB recurrence risk factors are rare.

Thus, we aimed to determine the TB recurrence rate after the completion of the first anti-TB chemotherapy, and to examine the risk factors for TB recurrence.

## 2. Materials and methods

### 2.1. Data source

This was a population-based, retrospective cohort study in which a nationally representative sample of the national health insurance beneficiaries in Korea between 2002–2013 was analyzed. The National Health Insurance Service-National Sample Cohort (NHIS-NSC) was established through stratified random sampling of 2.2% from the total Korean population enrolled in the NHIS, the single universal insurer in Korea from 2002–2013. The cohort comprises four datasets, including participants’ insurance eligibility, medical treatment, medical institutions, and general health examinations containing socio-economic, demographic variables, and information about all medical claims. The general health examination database contains results of health examination and information about lifestyle and behaviors from questionnaires [12].

### 2.2. Study population

Patients with newly diagnosed TB were included in this study. The definition of TB included all the following: (1) International Classification of Disease-Tenth Revision, ICD-10 codes for TB (A15-A19), (2) prescription of at least two different medications, among isoniazid (INH), rifampicin (RIF), ethambutol (EMB), and pyrazinamide (PZA), for longer than 28 days within a year. To adjust for potential confounding factors, we then excluded patients who had ICD-10 codes for TB or a prescription history of any anti-TB medications during a washout period of two years (2002–2003).

### 2.3. Outcome and covariates

The primary outcome of this study was TB recurrence, which can be either re-activation with the same strain (i.e., relapse), or re-infection with a new strain [13]. We defined TB recurrence as the second diagnosis of TB documented 6 months after completing the first TB treatment. The completion of treatment was defined as having received ≥ 90% of the recommended dose over a 6-month regimen within 9 months, or 9-month regimen within 12 months, respectively.

Data for sociodemographic characteristics, comorbidities and other clinical characteristics were obtained from the NHIS database. Socio-demographic characteristics, including sex (male or female), age (18 age groups: infants under 1 year, ages 1–4, 5-year age groups between 5 and 79, and 80 years and above), and income (Q1, Q2, Q3, Q4, or Q5) were analyzed. The NHIS data provides information of income deciles based on the insurance contribution, which is imposed proportionally to monthly income [12]. In the study, we categorized patients into five income categories, where Q1 and Q5 indicate the lowest and highest, respectively. Comorbidities include diabetes, chronic respiratory diseases, malignant diseases, long-term steroid use, human immunodeficiency virus/acquired immunodeficiency syndrome (HIV/AIDS), organ transplant, chronic kidney disease, abnormal weight loss or Crohn’s disease. We defined each comorbidity with diagnosis records at least twice during one year prior to the index date to the end of follow-up. We obtained the following information from the general health examinations dataset: Body Mass Index (BMI < 18.5 or 18.5 ≤ kg/m^2^), fasting blood sugar (< 110 or 110 ≤ mg/dL), history of smoking (never/former or current) and alcohol use (0–2 days, 3–5 days, or 6–7 days per week). Individuals may not have the results of health examinations for every year, depending on the types of insurance subscription [14]. Thus, the nearest values to the baseline year of each individual were used to minimize the loss of the study sample.

### 2.4. Statistical analysis

Baseline characteristics of individuals with recurrent TB were compared with those without recurrence, using a two-tailed student’s t-test for continuous variables, and a χ2 test for categorical variables. Cox regression analysis was performed on risk factors of TB recurrence. We produced hazard ratios (HR) and 95% confidence interval (CI) to determine whether a predefined set of variables are risk factors for TB recurrence. In addition to the main analysis, we also performed subgroup analysis among samples with health examination data for associations between TB recurrence and BMI and fasting blood glucose, stratified by sex, age and history of diabetes. All analyses were conducted using SAS software, version 9.3 (SAS Institute Inc., Cary, NC, USA) and a p-value < 0.05 was considered to indicate statistical significance.

### 2.5. Ethical approval

This study was conducted according to the 2008 Declaration of Helsinki and was approved by the independent Institutional Review Board of Yonsei University Health System (IRB number: 4-2019-0917). No written informed consent was required, as patient records/information was anonymized and de-identified prior to analysis. The need of an informed consent form was waived by the Institutional Review Board of Yonsei University Health System.

## 3. Results

**Fig 1:**
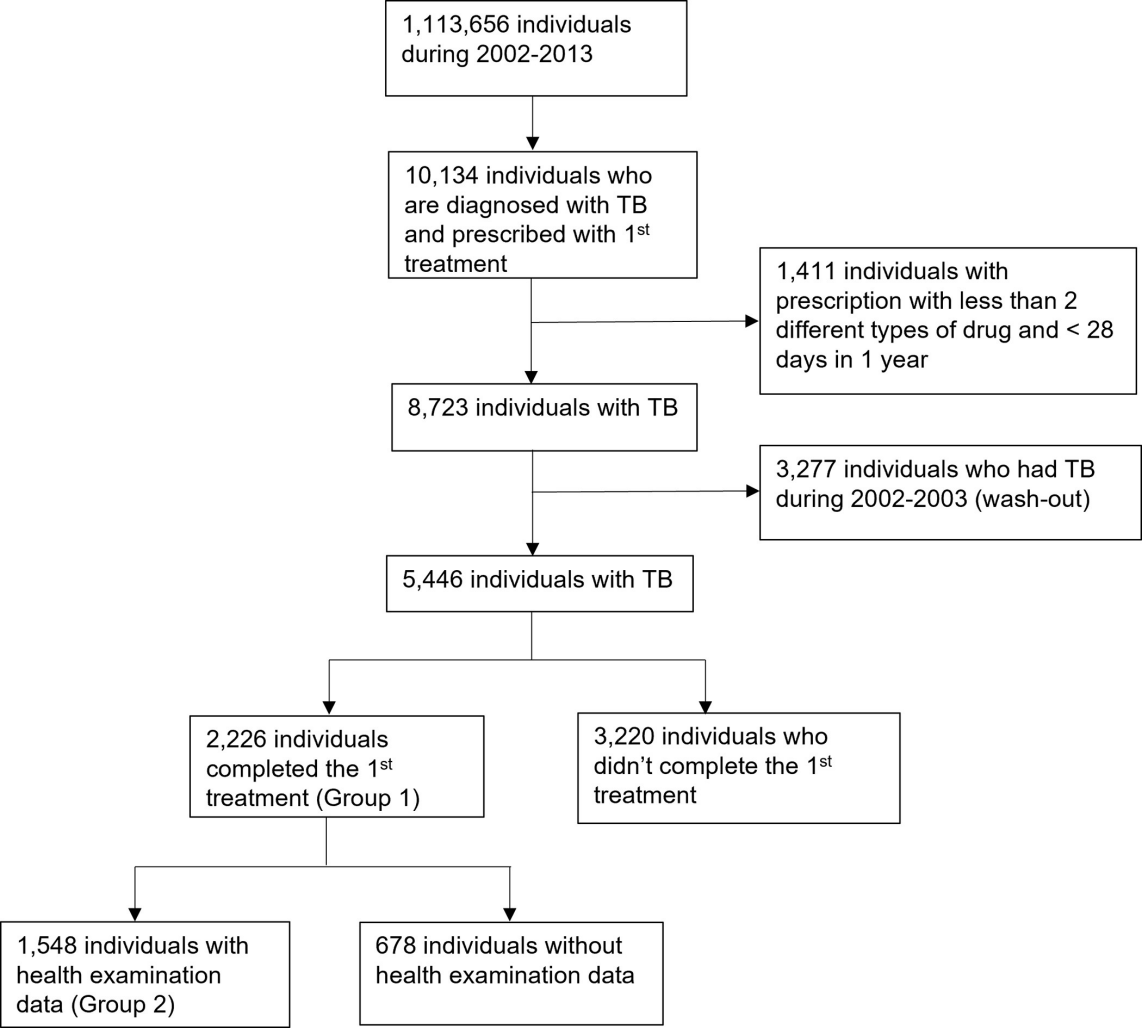
Flow diagram of the study population.

Among 1,113,656 individuals who were initially enrolled, 8,723 satisfied the definition of TB between 2002 and 2013. After excluding a previous history of ICD-10 codes, or the prescription, 5,446 participants with newly diagnosed TB were identified (Fig 1). Among these individuals, 2,226 participants completed the TB treatment based on the definition (Group 1). Of those, 1,548 (Group 2) had health examination data from the general health examinations dataset (Fig 1). We analyzed and reported the two groups separately. The groups constituted 12,094.4 and 8,530.4 person-years of follow-up, respectively.

### 3.1. Baseline characteristics and TB recurrence

Table 1 shows the general characteristics of the study participants. Group 1 represents all 2,226 people who completed the first anti-TB treatment. They included 1,271 (57.1%) males and 955 (42.9%) females, and their median age was ranged between 45–49 years old. The lower income groups (Q1, Q2) represent 754 (33.9%) participants and the higher income groups (Q4, Q5) represent 1,002 (45.0%). Chronic respiratory disease, including asthma, chronic obstructive pulmonary disease, and bronchiectasis was the most common co-morbidity (32.7%). Diabetes was also a prevalent co-morbidity (21.1%), while 1,036 (46.5%) did not have any of the specified co-morbidities. Among the 1,548 participants in Group 2 with heath examination data, males constituted 896 (57.9%) and the prevalence of chronic respiratory disease and diabetes were 34.2% and 22.9%, respectively. As shown in Table 1, 187 (12.1%) participants in Group 2 were underweight and 421 (27.2%) were current smokers.

**Table 1:** Characteristics of study population.

Variables	Group 1		Group 2	
	(All)		(with health exam data)	
	N	%	N	%
**Total**	2,226	100.0	1,548	100.0
**Panel A: demographic characteristics**				
**Sex**				
Male	1,271	57.1	896	57.9
Female	955	42.9	652	42.1
**Age**	9.6	3.8	10.0	3.5
< 20	122	5.5	22	1.4
20–29	467	21.0	291	18.8
30–39	421	18.9	307	19.8
40–49	350	15.7	266	17.2
50–59	300	13.5	235	15.2
60–69	270	12.1	220	14.2
≥70	296	13.3	207	13.4
**Income**				
Q1	347	15.6	233	15.1
Q2	407	18.3	274	17.7
Q3	470	21.1	338	21.8
Q4	462	20.8	334	21.6
Q5	540	24.3	369	23.8
**Comorbidities**				
** Chronic respiratory disease**	729	32.7	529	34.2
** Diabetes**	470	21.1	355	22.9
** Malignant disease**	318	14.3	229	14.8
** Long-term steroid use**	215	9.7	156	10.1
** Others**	51	2.3	35	2.3
**Panel B: clinical characteristics from health screening data**				
**BMI** (kg/m2)				
< 18.5			187	12.1
18.5 ≤			1,361	87.9
**Fasting blood sugar** (mg/dL)				
< 110			1,277	82.5
110 ≤			271	17.5
**Smoking**				
Never or former smoker			1,127	72.8
Current smoker			421	27.2
**Drinking**				
0–2 days /week			1360	87.9
3–5 days /week			169	10.9
6–7 days /week			19	1.2

TB: Tuberculosis. TB recurrence refers to the recurrence of TB 6 months after the end of the first treatment. Chronic respiratory disease includes asthma, chronic obstructive pulmonary disease and silicosis. Other comorbidities include AIDS/HIV, transplant, chronic kidney disease, abnormal weight loss and Crohn’s diseases. Income level is defined using National Health Insurance premiums and categorized from low(Q1) to high (Q5) levels.

### 3.2. Recurrence of TB after treatment completion

**Fig 2:**
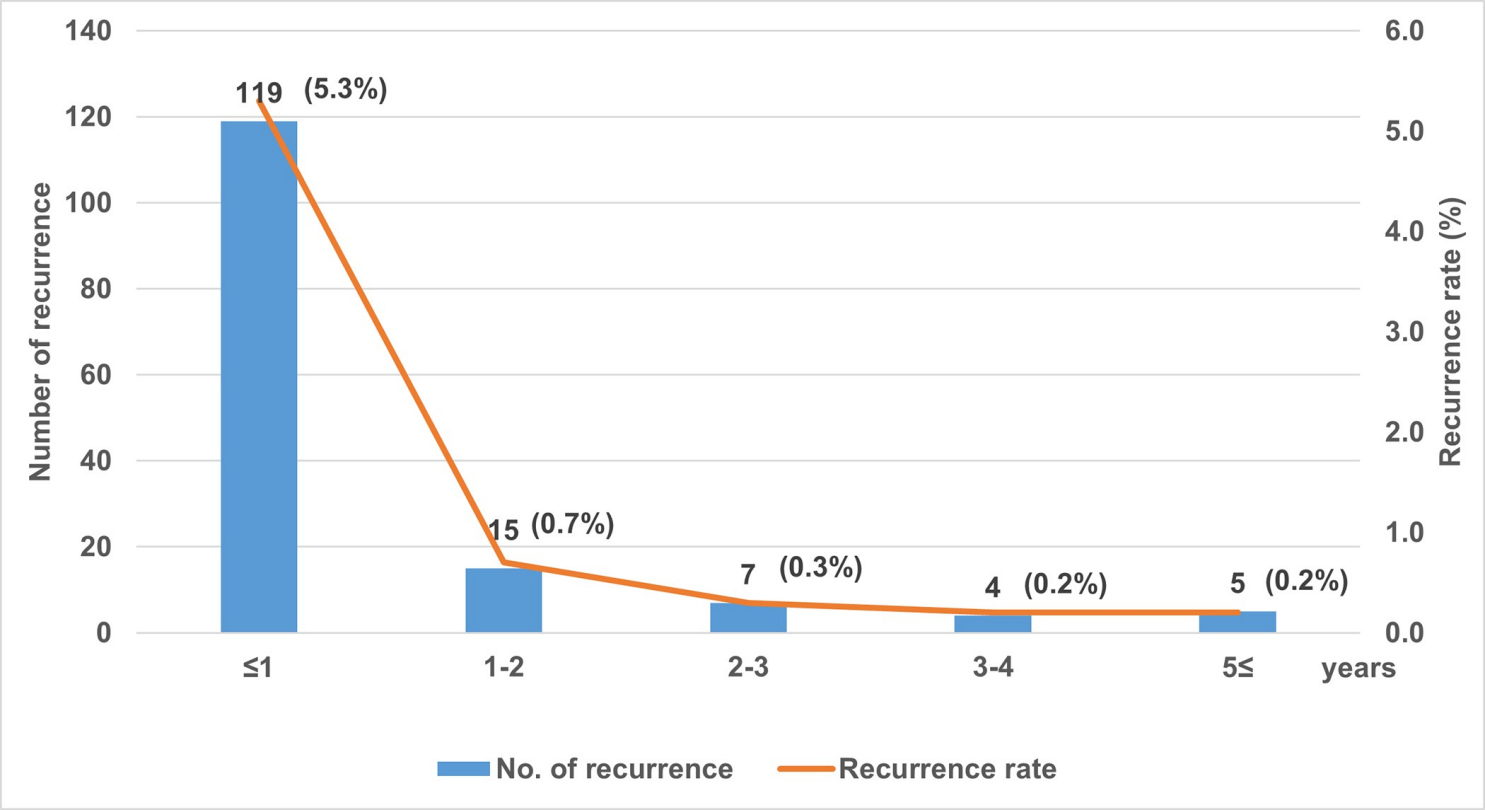
Recurrence of tuberculosis by years after completing the first treatment.

Among all 2,226 participants, 150 (6.7%) of them had recurrence of TB during the follow-up period (Table 2). An incidence rate of 12.4 per 1000 person-years was determined. The number of recurrent cases decreased yearly: 119 (5.3%) within 1 year, 15 (0.7%) in 1–2 years, 7 (0.3%) in 2–3 years, 4 (0.2%) in 3–4 years and 5 (0.2%) in over 5 years. Overall, these data show that 89% of recurrent cases occurred within the first 2-year period (Fig 2).

**Table 2:** Characteristics of study population by TB recurrence.

Variables	Group 1					Group 2				
	(All)					(with health exam data)				
	TB recurrence -		TB recurrence +		P-value	TB recurrence -		TB recurrence +		P-value
	N	%	N	%		N	%	N	%	
**Total**	2,076	93.3	150	6.7		1,451	93.7	97	6.3	
**Panel A:demographic characteristics**										
**Sex**					0.014					0.012
Male	1,171	92.1	100	7.9		828	92.4	68	7.6	
Female	905	94.8	50	5.2		623	95.6	29	4.4	
**Age**					0.102					0.860
< 20	106	86.9	16	13.1		20	90.9	2	9.1	
20–29	444	95.1	23	4.9		278	95.5	13	4.5	
30–39	392	93.1	29	6.9		286	93.2	21	6.8	
40–49	327	93.4	23	6.6		249	93.6	17	6.4	
50–59	278	92.7	22	7.3		221	94.0	14	6.0	
60–69	252	93.3	18	6.7		205	93.2	15	6.8	
≥70	277	93.6	19	6.4		192	92.8	15	7.2	
**Income**					0.039					0.010
Q1	328	94.5	19	5.5		223	95.7	10	4.3	
Q2	367	90.2	40	9.8		246	89.8	28	10.2	
Q3	447	95.1	23	4.9		325	96.2	13	3.8	
Q4	428	92.6	34	7.4		309	92.5	25	7.5	
Q5	506	93.7	34	6.3		348	94.3	21	5.7	
**Comorbidities**										
** Chronic respiratory disease**	684	93.8	45	6.2	0.458	498	94.1	31	5.9	0.635
** Diabetes**	444	94.5	26	5.5	0.240	338	95.2	17	4.8	0.191
** Malignant disease**	302	95.0	16	5.0	0.190	221	96.5	8	3.5	0.061
** Long-term steroid use**	193	89.8	22	10.2	0.032	144	92.3	12	7.7	0.438
** Others**	22	43.1	2	3.9	0.750	33	94.3	2	5.7	0.892
**BMI** (kg/m2)										0.019
< 18.5						168	89.8	19	10.2	
18.5 ≤						1,283	94.3	78	5.7	
**Fasting blood sugar** (mg/dL)										0.267
< 110						1,201	94.0	76	6.0	
110 ≤						250	92.3	21	7.7	
**Smoking**										0.420
Never or former smoker						1,060	94.1	67	5.9	
Current smoker						391	92.9	30	7.1	
**Drinking**										0.068
0–2 days /week						1282	94.3	78	5.7	
3–5 days /week						152	89.9	17	10.1	
6–7 days /week						17	89.5	2	10.5	

TB: Tuberculosis. TB recurrence refers to the recurrence of TB 6 months after the end of the first treatment. Chronic respiratory disease includes asthma, chronic obstructive pulmonary disease and silicosis. Other comorbidities include AIDS/HIV, transplant, chronic kidney disease, abnormal weight loss and Crohn’s diseases. Income level is defined using National Health Insurance premiums and categorized from low(Q1) to high (Q5) levels.

Table 2 shows the different characteristics between the groups with and without TB recurrence. Male exhibited a higher incidence of TB recurrence (7.9%) than females (5.2%). We found a statistically different distribution for sex (p = 0.014) and income (p = 0.039) between those with or without TB recurrence. Among the 1,548 participants in Group 2 with health examination data, 97 (6.3%) had showed a recurrence of TB. As shown in Table 2, people who were underweight had more frequent TB recurrence (10.2% vs 5.7%, p = 0.019). A similar pattern was observed in the frequent drinking group (p = 0.068).

### 3.3. Risk factors associated with TB recurrence

Table 3 summarizes risk factors for TB recurrence. In Group 1, we found sex was significantly associated with TB recurrence, and that males were at a higher risk than females (adjusted HR [aHR] = 1.60, at a 95% CI, range: 1.14–2.25). We found inconsistent results for income level, showing that only Q2 was significantly higher than Q5 (Q2: aHR = 1.66, at a 95% CI, range: 1.05–2.64). In Group 2, we found that males (aHR = 1.81, at a 95% CI, range: 1.10–2.94) and older ages (aHR = 1.07, at a 95% CI, range: 1.00–1.14) were significantly associated with an increased HR for TB recurrence. People who were underweight (HR = 1.74, at a 95% CI, range: 1.06–2.88) and frequent alcohol drinkers (3–5 days /week unadjusted HR = 1.81, at a 95% CI, range: 1.07–3.05) were also at higher risk for TB recurrence. BMI remained statistically significant after adjusting for other risk factors (aHR = 1.92, at a 95% CI, range: 1.15–3.20).

**Table 3:** Risk factors associated with TB recurrence.

Variables	TB recurrence +																	
	Group 1									Group 2								
	(All, n = 2,226)									(with health exam data, n = 1,548)								
	Unadjusted				Adjusted				*p- value*	Unadjusted				Adjusted				*p- value*
	HR	95%CI			HR	95%CI				HR	95%CI			HR	95%CI			
**Panel A: general characteristics**																		
**Gender** (ref = female)																		
Male	1.54	(1.10	-	2.16)	1.60	(1.14	-	2.25)	0.007	1.76	(1.14	-	2.72)	1.81	(1.11	-	2.94)	0.017
**Age**	1.00	(0.96	-	1.05)	1.03	(0.98	-	1.08)	0.330	1.04	(0.98	-	1.10)	1.07	(1.00	-	1.14)	0.048
**Income** (ref = Q5)									0.020									0.003
Q1	0.89	(0.51	-	1.56)	0.87	(0.50	-	1.53)		0.72	(0.34	-	1.53)	0.68	(0.32	-	1.45)	
Q2	1.61	(1.02	-	2.54)	1.66	(1.05	-	2.64)		1.86	(1.06	-	3.27)	1.96	(1.10	-	3.48)	
Q3	0.76	(0.45	-	1.28)	0.75	(0.44	-	1.27)		0.64	(0.32	-	1.29)	0.61	(0.30	-	1.22)	
Q4	1.16	(0.72	-	1.86)	1.16	(0.72	-	1.86)		1.28	(0.72	-	2.29)	1.24	(0.69	-	2.24)	
**History of chronic respiratory disease** (ref = no)	0.85	(0.60	-	1.21)	0.82	(0.56	-	1.18)	0.281	0.88	(0.58	-	1.35)	0.76	(0.48	-	1.20)	0.237
**History of diabetes** (ref = no)	0.78	(0.51	-	1.19)	0.73	(0.46	-	1.15)	0.175	0.71	(0.42	-	1.20)	0.54	(0.30	-	0.98)	0.044
**History of Malignant disease** (ref = no)	0.74	(0.44	-	1.25)	0.74	(0.43	-	1.27)	0.279	0.52	(0.25	-	1.08)	0.45	(0.22	-	0.95)	0.035
**History of long-term steroid use** (ref = no)	1.61	(1.02	-	2.53)	1.75	(1.10	-	2.79)	0.018	1.26	(0.69	-	2.31)	1.30	(0.69	-	2.43)	0.415
**History of other comorbidities** (ref = no)	1.14	(0.42	-	3.07)	0.91	(0.33	-	2.48)	0.848	0.90	(0.22	-	3.65)	0.78	(0.19	-	3.24)	0.736
**Panel B: clinical characteristics from health screening dataset**																		
**BMI** (kg/m2)																		0.012
< 18.5										1.74	(1.06	-	2.88)	1.92	(1.15	-	3.20)	
18.5 ≤										1.00				1.00				
**Fasting blood sugar** (mg/dL)																		0.090
< 110										1.00				1.00				
110 ≤										1.36	(0.84	-	2.20)	1.60	(0.93	-	2.77)	
**Smoking**																		0.469
Never or former smoker										1.00				1.00				
Current smoker										1.19	(0.78	-	1.83)	0.84	(0.51	-	1.36)	
**Drinking**																		0.250
0–2 days /week										1.00				1.00				
3–5 days /week										1.81	(1.07	-	3.05)	1.60	(0.92	-	2.79)	
6–7 days /week										1.79	(0.44	-	7.30)	1.30	(0.31	-	5.41)	

TB: Tuberculosis. TB recurrence refers to the recurrence of TB 6 months after the end of the first treatment. Chronic respiratory disease includes asthma, chronic obstructive pulmonary disease and silicosis. Other comorbidities include AIDS/HIV, transplant, abnormal weight loss and Crohn’s diseases. Income level is defined using NHI premiums, and categorized from low(Q1) to high (Q5) levels.

### 3.4. Association of BMI and TB recurrence

**Fig 3:**
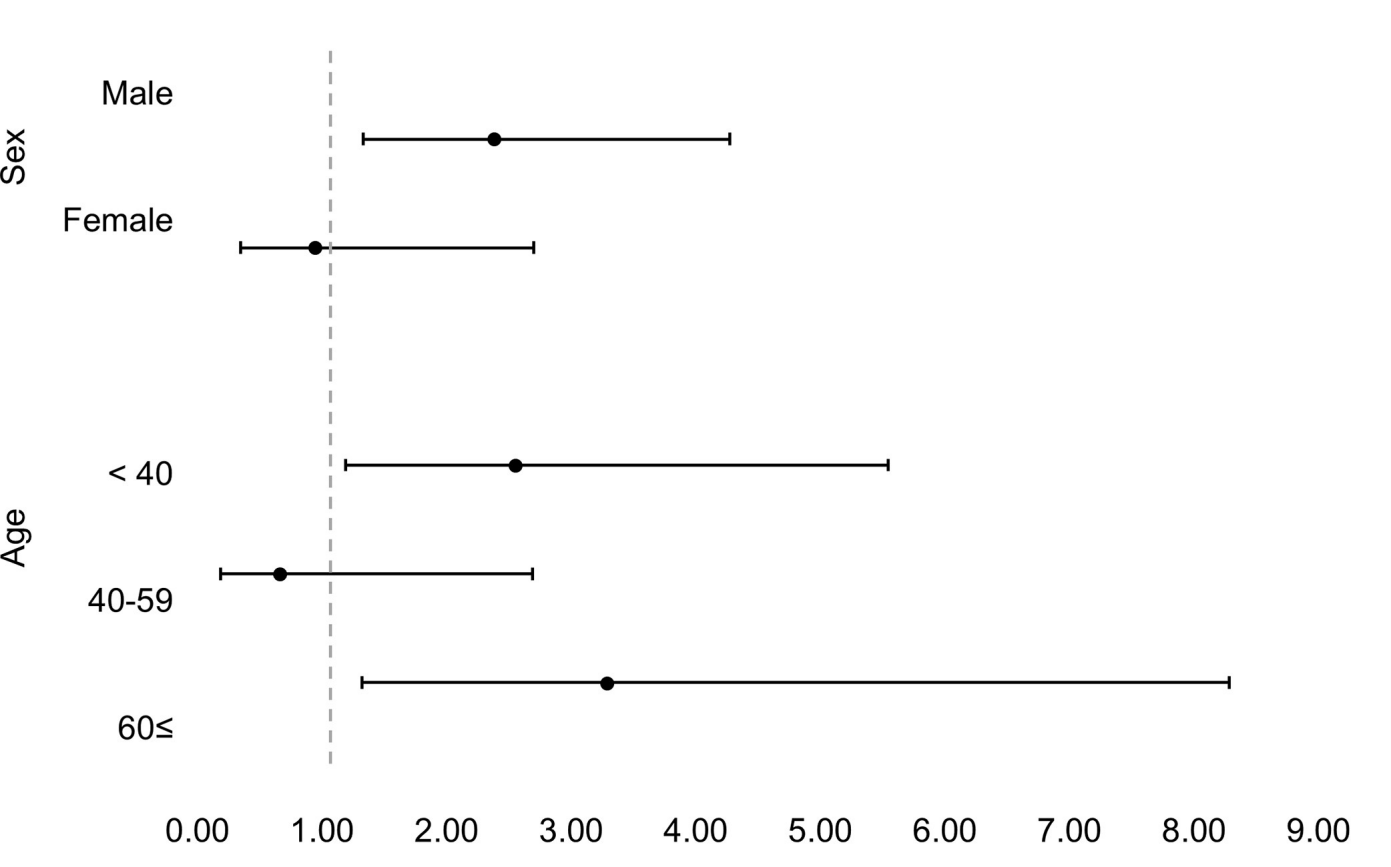
Results of subgroup analysis for association between TB recurrence and BMI, stratified by sex and age among subjects with health exam data (BMI < 18.5 vs 18.5 ≤). (Abbreviation: BMI, Body Mass Index, TB, tuberculosis).

We performed a subgroup analysis to examine the association between BMI and TB recurrence, stratified by age and sex (Fig 3). The results represent those underweight patients who were at a higher risk of TB recurrence when compared to normal or overweight people among those who are male (aHR = 2.34, at a 95% CI, range: 1.30–4.24), younger than 40 years old (aHR = 2.52, at a 95% CI, range: 1.15–5.51) or older than 60 years old (aHR = 3.25, at a 95% CI, range: 1.28–8.25).

## 4. Discussion

We examined the risk factors associated with TB recurrence, among people who had completed their first treatment of TB, using a nationwide cohort study in Korea. We found an incidence rate of 6.7% (22.6 per 1000 person-years) for TB recurrence, and that being male and being underweight were significant risk factor. Being underweight had a particular impact on TB recurrence in participants who were male and younger than 40 years old or older than 60 years old.

The rate of TB recurrence varies across different regions. Using individual data from recent randomized controlled trials, research estimates a 5.6% relapse rate within 18–24 months of follow-up after a WHO-standard 6-month regimen for pulmonary TB [15]. Other studies have reported a rate of 8–10% using longitudinal study settings [1, 16]. In Korea, Jo *et al* have reported a 1.9% 1-year relapse rate after 6-month of treatment for pulmonary TB in a tertiary hospital based retrospective cohort [17]. Additionally, Lee *et al* reported a 5-year re-reported rate of 9.7% regardless of treatment outcomes for all registered TB cases in a national system in 2005 [18]. In our study, the overall recurrence rate was 6.7%. The highest recurrence rate was 5.3% in the first year and consistently declined afterwards. This is in accordance with previous studies that show most recurrences occur within 1–2 years of successful treatment completion [16, 18–20]. In Korea, Lee *et al* have reported that 43% of the total re-reported cases occurred during the first year of follow-up [18]. TB patients are known to be at risk for recurrence of TB even after they were cured or potential sequelae, thus clinicians could follow up after treatment for a minimum of 12 months [21].

Various factors for increasing the risks of TB recurrence have been identified, including demographic characteristics (e.g., sex [18] and age [22, 23]) socio-economic determinants (e.g., low income, low education level and unemployment [24, 25]), behavioral factors (e.g., smoking [23] and drinking [26]), clinical characteristics (e.g., malnutrition and low body weight [27]), and comorbidities (e.g., HIV infection [28, 29] and diabetes [30, 31]).

In accordance with prior studies, we found that being male and older were associated with an increased likelihood of recurrent TB. Age is a well-established risk factor for TB recurrence. As discussed in previous studies, older age is likely to be associated with a weak immune system, other underlying disease, and adverse drug reaction, all of which may lead to reactivation of the strain [32–34].

Another key finding of this study is that low BMI increases the risk of TB recurrence. Earlier studies have reported evidence on weight and BMI as predictors for TB development [17, 27, 35]. These factors are often used as markers of nutritional status, indicating that being underweight or having a low BMI are often correlated with undernutrition, and thus, a higher risk of developing TB. Moreover, being underweight and having a low BMI is associated with severity of disease, a poor treatment response [36], relapse [27] and mortality [37]. From these results, we also found lower BMI is significantly associated with the increased risks of TB recurrence in males, those under 40 or over 60 years old. This suggests that across populations with different levels of risks for other health problems, BMI may be used as an independent risk factor for TB recurrence.

The study has several limitations. As we used cohort data collected from the insurance claims database, there is information we could not retrieve. First, we could not analyze the clinical factors of TB, such as disease severity (cavity and AFB smear) and treatment response (culture positivity at 2 months). Moreover, we could not consider severity of comorbidities, which may be associated with TB recurrence. Second, information on drinking and smoking is collected from self-reported questionnaires, which may lead to potential underestimated prevalence [38]. Third, the completion rate of anti-TB treatment in our study was lower than reported data. The study population might be selected with bias focused on standard 6 –or 9-month regimen completer. Fourth, prescription information in NHIS data alone may not be sufficient to measure duration of medication treatment accurately. In this study, we assumed that people correctly comply to medication as prescribed. Fifth, we were not able to control for certain confounding characteristics that might have been associated with TB recurrence, such as socio-economic factors, including living area, occupation, and education. Previous studies have discussed education and occupation as factors associated with TB prevalence and treatment outcomes [39]. In addition to individual characteristics, epidemiological features of living area are known to have an effect [28].

Despite these limitations, we were able to demonstrate risk factors for TB recurrence using a nationally representative sample and increase homogeneity by identifying newly diagnosed TB patients during the observational period. This study provides evidence on the risk factors of TB recurrence in Korea, where TB is a considerable burden.

## 5. Conclusion

In summary, we examined risk factors for TB recurrence following successful treatment completion. Our findings suggest that most of TB recurrences occur within 1–2 years after completing treatment, and as for risk factors, being male, being older in age, and having a low BMI are significantly associated with recurrence. Further studies are required to expand current understanding of TB recurrence and identify vulnerable groups. This will aid the establishment of strategies for more timely and effective TB prevention and treatment.

## Abbreviations

aHR: Adjusted Hazard Ratio
AIDS: Acquired immunodeficiency syndrome
BMI: Body Mass Index
CCI: Charlson’s Comorbidity Index
CI: Confidence Interval
EMB: Ethambutol
HIV: Human immunodeficiency virus
HR: Hazard Ratio
ICD: International Standard Classification of Disease
INH: Isoniazid
NHIS: National Health Insurance Service
NHIS-NSC: National Health Insurance Service-National Sample Cohort
PZA: Pyrazinamide
RIF: Rifampicin
TB: Tuberculosis
